# Superconducting imprint of magnetic textures in ferromagnets with perpendicular magnetic anisotropy

**DOI:** 10.1038/s41598-021-99963-w

**Published:** 2021-10-21

**Authors:** A. Sander, G. Orfila, D. Sanchez-Manzano, N. Reyren, M. A. Mawass, F. Gallego, S. Collin, K. Bouzehouane, K. Höflich, F. Kronast, F. Grilli, A. Rivera-Calzada, J. Santamaria, J. E. Villegas, S. Valencia

**Affiliations:** 1 Unité Mixte de Physique, CNRS, Thales, Université Paris-Saclay, 91767 Palaiseau, France; 2 GFMC. Dept. Fisica de Materiales, Facultad de Fisica, Universidad Complutense, 28040 Madrid, Spain; 3 Helmholtz-Zentrum Berlin für Materialien und Energie, Albert-Einstein-Str. 15, 12489 Berlin, Germany; 4 Karlsruher Institut für Technologie, Institut für Technische Physik, 76344 Eggenstein-Leopoldshafen, Germany; 5 Present Address: Ferdinand-Braun-Institut GMbH Leibnitz-Institut für Höchstfrequenztechnik, Gustav-Kirchhoff-Str. 4, 12489 Berlin, Germany

**Keywords:** Materials science, Condensed-matter physics, Materials for devices, Condensed-matter physics, Ferromagnetism, Magnetic properties and materials, Spintronics, Superconducting properties and materials, Surfaces, interfaces and thin films

## Abstract

Research on proximity effects in superconductor/ferromagnetic hybrids has most often focused on how superconducting properties are affected—and can be controlled—by the effects of the ferromagnet’s exchange or magnetic fringe fields. The opposite, namely the possibility to craft, tailor and stabilize the magnetic texture in a ferromagnet by exploiting superconducting effects, has been more seldom explored. Here we show that the magnetic flux trapped in high-temperature superconducting YBa_2_Cu_3_O_7-δ_ microstructures can be used to modify the magnetic reversal of a hard ferromagnet—a cobalt/platinum multilayer with perpendicular magnetic anisotropy—and to imprint unusual magnetic domain distributions in a controlled manner via the magnetic field history. The domain distributions imprinted in the superconducting state remain stable, in absence of an external magnetic field, even after increasing the temperature well above the superconducting critical temperature, at variance to what has been observed for soft ferromagnets with in-plane magnetic anisotropy. This opens the possibility of having non-trivial magnetic configuration textures at room temperature after being tailored below the superconducting transition temperature. The observed effects are well explained by micromagnetic simulations that demonstrate the role played by the magnetic field from the superconductor on the nucleation, propagation, and stabilization of magnetic domains.

## Introduction

Conventional (singlet) superconductivity and ferromagnetism are antagonistic phenomena which rarely coexist in bulk materials^1,2^. While conventional superconductivity requires the antiparallel alignment of the electron’s spin in (singlet) Cooper pairs, ferromagnetism favors their parallel arrangement. This has motivated the investigation of superconductor/ferromagnet (SC/FM) interactions in hybrids such as multilayers where superconductivity and ferromagnetism reside in different layers. Many of these studies have focused on investigating how superconducting properties are affected or can be manipulated by the exchange interaction or the magnetic stray fields from the ferromagnet. This includes, for instance, effects on the superconducting critical temperature T_C_ (Refs.^3,4^) or on the critical currents^5–8^. The effect of SC stray fields on the FM domain textures has been addressed less often. Recent reports, both theoretical and experimental, have shown the possibility to use SCs to craft non-trivial magnetization textures, stable at zero magnetic field, in adjacent ferromagnets with lateral dimensions ranging from the nano^9–11^ to the micro-scale^12–14^. Such effect stems from the inhomogeneous magnetic stray field from the superconductor $\overrightarrow{H}$_SC_ (*x*,*y*), which is created either directly by superconducting vortices^9–11^ or by the circulation of screening supercurrents^12–14^ and the penetration and pinning of magnetic flux quanta^15,16^. The stray magnetic field arising from screening supercurrents, which depends on magnetic field history^16,17^ and on the geometry of the SC structure^18,19^, can modify the magnetic domains distribution of an overlying FM thin film^19–23^. Indeed, recent experiments exploit this SC/FM interaction to characterize superconducting film properties using the ferromagnetic layer as a magneto-optically active material^24^, or to imprint magnetic unusual spin textures such as the recently reported skyrmion-like magnetic domain configurations in soft ferromagnets^10^.

Most of the research on the imprint of magnetic domains by means of superconductor stray fields has focused on YBa_2_Cu_3_O_7-δ_/FM hybrids with the FM being a thin layer with in-plane magnetic anisotropy (IMA), namely Co_40_Fe_40_B_20_ (Refs.^25,26^) or Fe_20_Ni_80_ (Py) (Refs.^10,14,27,28^). In these systems the imprinted domain´s magnetization, essentially confined in plane^10,14,28^, is only stable well below T_C_ (Ref.^28^).

In this work we investigate the superconducting imprint and stabilization, in the absence of an external magnetic field, of magnetic textures in hybrid SC/FM structures with SC being the high temperature superconductor YBa_2_Cu_3_O_7−δ_ (YBCO) and FM a ferromagnetic multilayer with strong perpendicular magnetic anisotropy (PMA), namely a Co/Pt multilayer. We show that the superconducting field generated by micrometric YBCO squares and discs modifies the magnetization reversal at a local scale. While in the normal state of YBCO the switching of the local magnetization occurs randomly upon application of a sufficiently high out-of-plane external magnetic field *H*^z^_ext,_, in the superconducting state the magnetization switches at significantly lower *H*^z^_ext_ near the edges of the structure rather than at its center where nucleation occurs for larger fields, leading to a spatial distribution of switching fields whose symmetry is dictated by the SC structure’s geometry.

In the studied cases of SC squares and discs, that effect allows artificially imprinting concentric magnetic domain regions with antiparallel out-of-plane magnetization orientation. The relative size of these regions at remanence (*H*^*z*^_ext_ = 0) depends on the applied magnetic field history. Temperature dependent measurements demonstrate that the imprinted domain structure remains stable well above the superconducting transition temperature.

## Experiment

A 130 nm thick YBCO layer was deposited by means of d.c. magnetron sputtering on top of a (001)-oriented Nb-doped SrTiO_3_ substrate. YBCO growth conditions were optimized for epitaxial c axis growth. X-ray characterization and high-resolution microscopy ruled out the presence of a–b axis growth. CuO precipitates are typically observed at the surface of our samples (Supplementary Fig. S1). They result of the strongly oxidizing atmosphere of the high-pressure pure oxygen sputter deposition. The presence of CuO precipitates affects the (vortex) pinning landscape, and thus the vortex distribution of the Abrikosov vortex lattice. However, over the much longer length scale considered in the critical state model, the effect of those defects is averaged and boils down to a global increase of the critical current. Details of the deposition conditions are reported elsewhere^3^. Superconducting YBCO squares (13 µm side) and discs (13 µm diameter) were defined by means of optical lithography and wet etching. After this etching procedure, a [Co (0.6 nm)/Pt (1 nm)] × 5 multilayer—grown on top of a 5 nm Pt buffer favoring the (1 1 1)-texture that produces a large PMA—was deposited over the entire sample surface using magnetron sputtering at room temperature.

**Figure 1:**
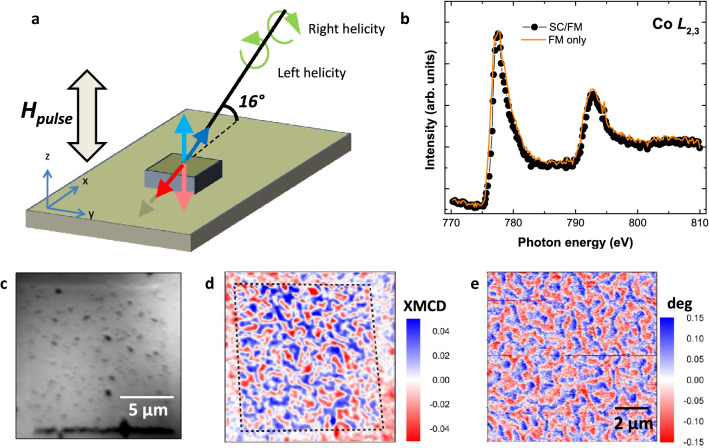
(**a**) Sketch of the experimental set-up. XMCD, measured as the normalized difference in absorption for incoming left and right helicity of circular polarized radiation, is proportional to the projection of the magnetization (light red and blue arrows) along the beam propagation direction (red and blue arrows). (**b**) Absorption spectrum obtained at the Co *L*_2,3_-edges for (Co/Pt)_5_ on top (dots) and around the SC structure (line). (**c,d**) XAS and XMCD images obtained at Co *L*_3_-edge (777.55 eV) at 50 K after a demagnetization process at 140 K. (**e**) MFM phase image showing similar magnetization pattern.

To investigate the role of superconducting stray fields on the magnetic domain configuration of the Co/Pt multilayer we imaged the domains distribution at remanence after out-of-plane magnetic field pulses *H*^*z*^_pulse_ and compared the results for experiments above and below T_C_. The magnetic domain pattern on the FM multilayer was imaged by means of photoemission electron microscopy (PEEM) using X-ray magnetic circular dichroism (XMCD) as magnetic contrast mechanism^29,30^ (Fig. 1a), see “Methods” section.

## Results

Figure 1b depicts the averaged Co *L*_3,2_ X-ray absorption spectrum (XAS) obtained on FM film deposited on a SC disc structure (dots). The spectral shape is alike to that measured at (Co/Pt)_5_ regions without SC underneath (line) as well as to that reported for metallic Co (Ref.^31^). Figure 1c,d depict the XAS (σ^-^ + σ^+^) and XMCD ((σ^–^ – σ^+^)/(σ^-^ + σ^+^)) images, respectively, obtained at 50 K for a square structure after a demagnetization process at 140 K followed by a zero-field cooling procedure. Blue (positive XMCD) and red (negative XMCD) colored regions in Fig. 1d, correspond to magnetic domains with out-of-plane magnetization direction pointing upwards and downwards, respectively (see Fig. 1a). We note the presence of a region ca. 1 µm wide around the hybrid YBCO/(Pt/Co)_5_ structure with depressed XMCD signal. Its origin is likely to be related to some rest of resist from the photolithographic process remaining at the edges. The surface of the YBCO structure is decorated, as typically found in thin YBCO films, by the presence of CuO precipitates spaced several microns (Fig. 1c). The YBCO grows around screw dislocations which for the thickness range of these experiments give rise to micron size square pyramids with flat and continuous terraces, see Supplementary Fig. S1. The good coverage of these terraces by the deposition of the FM layer gives rise to large magnetic domains (Fig. 1d, Sect. 1 of Supplementary Information for details), while the superconducting properties are preserved (not shown). The magnetic domain pattern imaged by XMCD-PEEM is similar to that observed by means of magnetic force microscopy (MFM) on a Co/Pt multilayer with the same structure as the one used for the PEEM experiments (see Fig. 1e) and characteristic of systems with perpendicular magnetic anisotropy^32^.

**Figure 2:**
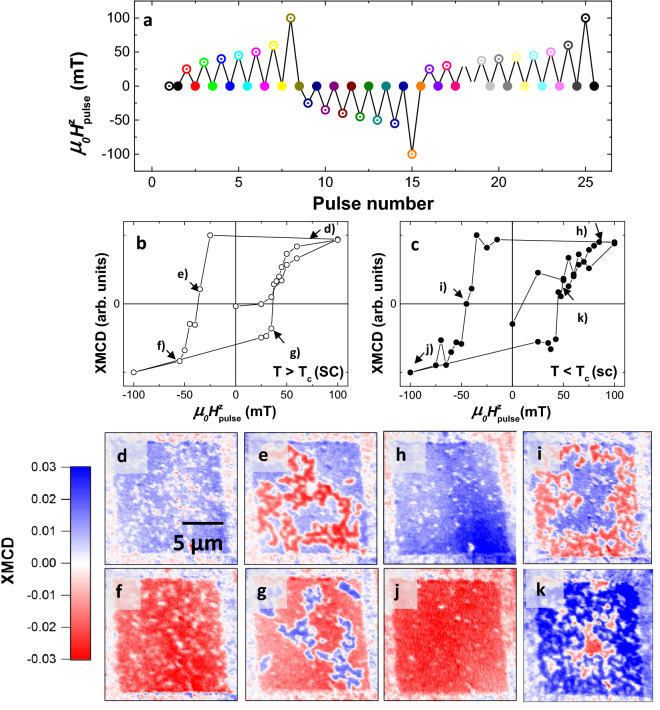
(**a**) Sketch of the magnetic pulse history followed at 120 K. XMCD imaging takes always place after pulse end (full dots). (**b,c**) XMCD averaged over the whole square structure vs *H*^z^_pulse_ within the normal (T > T_C_, (**b**)) and superconducting (T < T_C_, (**c**)) state of the YBCO square. (**d–g**) XMCD images at selected *H*^*z*^_*pulse*_ (see arrows in panel b) for T > T_C_. (**h–k**) XMCD images at selected *H*^*z*^_*pulse*_ (see arrows in (**c**)) for T < T_C_. Above T_C_ domain nucleation takes place at random positions while at for T < T_C_ it is confined at edges.

The magnetic domain structure of the (Co/Pt)_5_ multilayer on top of a 13 µm side YBCO square (130 nm thick) was measured as function of *H*^*z*^_pulse_ both, above (120 K) and below (50 K) the superconducting transition temperature of the YBCO film (T_C_≈ 90 K, see Ref.^3^). Magnetic field pulses of increasing amplitude *H*^*z*^_pulse_ were applied starting at µ_0_*H*^z^_pulse_ = 0 mT and finishing at µ_0_*H*^z^_pulse_ =  + 100 mT. Then, the amplitude was decreased stepwise down to − 100 mT and increased back to + 100 mT in an hysteresis loop sequence. An example of the typical pulses sequence is shown in Fig. 2a. The XMCD images were always obtained after each pulse i.e. at *H*^z^_ext_ = 0 (plain dots in Fig. 2a). We define the scalar field *H*^z^_s_(*x*,*y*) and the scalar ${H}_{s}^{z}$ as the pulse fields that allow the local and mean magnetization direction, respectively, to be switched at remanence. Notice that *H*^z^_s_(*x*,*y*) and *H*^z^_s_ are related to the coercive field, but they are not equivalent because the magnetic domain distribution created upon applying *H*^*z*^_pulse_ changes when the field is subsequently removed for imaging. Plots of the XMCD signal amplitude as function of *H*^*z*^_pulse_ results in hysteresis loops, in which the points XMCD = 0 indicate the switching field.

XMCD loops obtained with the procedure described above are depicted in Fig. 2b,c for T > T_C_ and T < T_C_ for the case in which the FM multilayer sits on top of an YBCO square. The XMCD data plotted in those graphs results from averaging the XMCD signal over the whole area of the hybrid SC/FM structure. We observe a sizable increase of the switching field of the mean magnetization with decreasing T (from µ_0_ ${H}_{s}^{z}$ *≈* 36 mT at T = 120 K to µ_0_ ${H}_{s}^{z}$ *≈* 44 mT at T = 50 K) concomitant with an increase of the fields necessary to saturate the loop (*H*_sat_). Analysis of the individual XMCD images allows a closer look into the processes involved in the nucleation and propagation of magnetic domains. Representative XMCD images of the ferromagnetic domain structure at saturation and close to ${H}_{s}^{z}$ (see arrows in Fig. 2b,c) are depicted in panels 2d–g and panels 2h–k for data obtained at T > T_C_ and T < T_C_, respectively.

In the normal state, i.e. for T > T_C_ the magnetic domain distribution nearby ${H}_{s}^{z}$ (Fig. 2e) and at the beginning of magnetization reversal (Fig. 2g) seems random, and there is no apparent correlation with the shape (square) of the SC structure. On the contrary, in the superconducting state (T < T_C_) the magnetic domains nucleate preferentially close or at the edges of the SC square, and propagate towards the center as │*H*^*z*^_pulse_│ increases. This is clearly seen in Fig. 2i,k, where we observe two concentric square regions: an inner region that retains the magnetization orientation set at saturation (Fig. 2h,j) and an external region where the magnetization is reversed. The differences in magnetization reversal and domain propagation can be clearly seen in Supplementary Videos S1 and S2 gathering the full set of XMCD vs *H*^*z*^_pulse_ images for T > T_C_ and T < T_C_, respectively.

**Figure 3:**
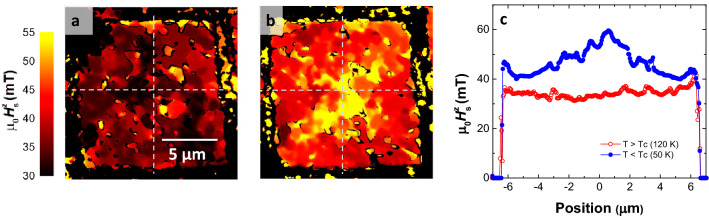
(**a,b**) 2D maps of the switching field above and below T_C_, respectively. Dashed lines indicate position along which 2 µm wide profiles have been obtained and averaged. The resulting line profile of *H*^z^_s_ are depicted in panel c) for T > T_C_ (red open dots) and T < T_C_ (blue filled dots). *H*^z^_s_ is almost constant within the normal state while it shows a clear position dependence for within the superconducting state.

In order to take a closer look into the magnetic domain nucleation process we use the XMCD vs *H*^*z*^_pulse_ image sets shown in Supplementary Videos S1 and S2 to determine the local switching fields *H*^z^_s_(*x*,*y*) over the SC/FM structure. The results are plotted as switching maps in Fig. 3a,b for T within the normal and superconducting state, respectively. Figure 3c shows the profiles of the switching field *H*^z^_s_ along the square axis (this is obtained by averaging *H*^z^_s_(*x*,*y*) over 2 µm-wide strips along the directions of the orthogonal square axis indicated by dashed lines in Fig. 3a,b. While the switching field at T > T_C_ (red symbols) stays almost constant at µ_0_*H*^z^_s_ ≈ 34 mT, we observe a clear spatial dependence at T < T_C_. In particular, the switching field is lower at the square edges where µ_0_*H*^z^_s_ *≈* 43 mT, and gradually increases as we move inside the square to reach a maximum µ_0_*H*^z^_s_ *≈* 60 mT at its center.

## Discussion

A first comment regards the possible influence of FM SC proximity interactions at the interface. In our case proximity effects have been disregarded for three main reasons: (i) The Co/Pt multilayers are deposited ex-situ on the YBCO film. This means that the YBCO surface is exposed to air and humidity before the magnetic structure is deposited. It is well known that under these conditions the YBCO surface is oxygen deficient and not superconducting (indeed, it becomes insulating), which dramatically reduces the interface transparency and quenches of the proximity effect^33,34^. (ii) As mentioned within the sample description it is important to notice that in our case there is a 5 nm Pt buffer layer in between the YBCO and the Co/Pt (8 nm) multilayer further preventing proximity effects. (iii) Finally, one has to note that PEEM is a surface technique (only the first 2–3 nm are probed), so that any possible proximity effect cannot be seen. We note also that the system’s properties and evidences at hand do not suggest that other long-range “proximity” scenarios, such as for example the giant proximity effect observed in all oxide YBCO/SrTiO_3_/La_2/3_Ca_1/3_MnO_3_ (Refs.^35,36^) could be responsible of our experimental observations. On the other hand, the effect of the stray field of the SC flux is robust towards details of the magnetic structure of the interface. For that reason, the effects of the SC state on the magnetic domain state of the FM/SC hybrids are discussed in terms of the stray field of the SC flux trapped in the YBCO dots.

To understand the differences in the nucleation and propagation of magnetic domains in the normal and the superconducting states of the YBCO we performed micromagnetic simulations with MuMax3^37–39^.

**Figure 4:**
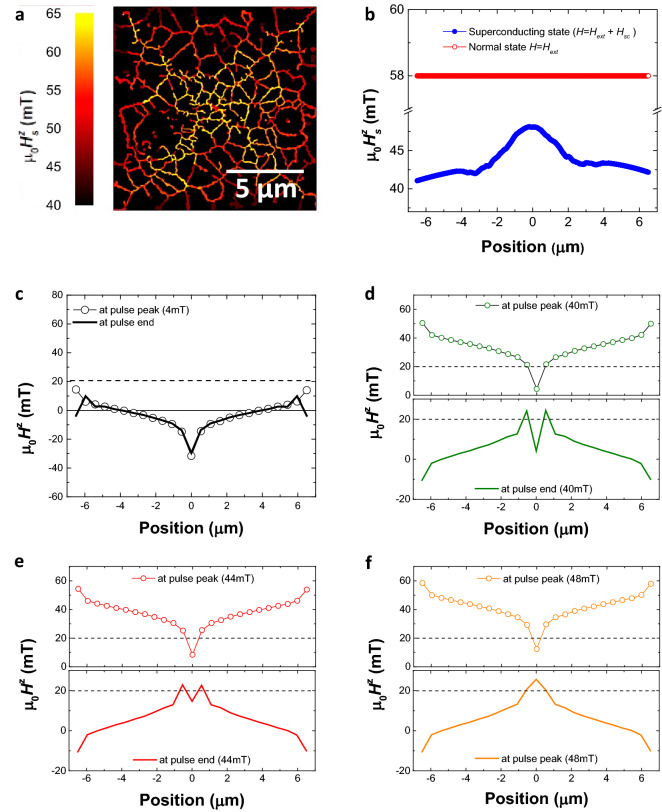
(**a**) 2D map of the computed switching field within the superconducting state. (**b**) Line profile of the computed switching field within the superconducting (blue dots) and normal (line) states. The profile is first averaged over 2-µm-wide lines passing through the center of the square along x and y, and then smoothed using a Savitzky-Golay filter. (**c–f**) Line profile of the z(x)-component of the effective field for various *H*^z^_pulse_ (4 mT, 40 mT, 44 mT and 48 mT) at pulse peak field (open dots) and at pulse end (line).

Calculations were performed by considering an isolated 13 × 13 µm^2^ square of 8-nm-thick ferromagnetic material with the effective parameters of the Co/Pt multilayer. To allow domain nucleation in these zero temperature simulations, a misalignment of 0.5° between the field and the normal to the sample plane was considered in all the simulations. In order to determine the switching field, we simulated a magnetic field history as for the experiments (see Sects. 2 and 3 of Supplementary Information for details). The magnetic state of the sample was simulated during and after each pulse, labelled ‘pulse peak’ and ‘pulse end’ respectively in Fig. 4c–f. The simulations were fed at every step with effective magnetic fields $\overrightarrow{H}$(*x*,*y*). In the YBCO normal state $\overrightarrow{H}$ = *H*^z^ = *H*^*z*^_pulse_ at ‘pulse peak’ and $\overrightarrow{H}$ = 0 at ‘pulse end’. In the YBCO superconducting state the effective magnetic field is modified by the stray field $\overrightarrow{H}$_SC_(*x*,*y*) generated by the superconductor at its surface, i.e. $\overrightarrow{H}$ = *H*^*z*^_pulse_ + $\overrightarrow{H}$_sc_ at ‘pulse peak’ and $\overrightarrow{H}$ = $\overrightarrow{H}$_sc_ at ‘pulse end’. Note that $\overrightarrow{H}$_sc_(*x*,y) depends on magnetic field history according to Bean’s critical state model^17^. In particular $\overrightarrow{H}$_sc_(*x*,*y*) under applied field (‘pulse peak’) and in zero applied field (‘pulse end’) are different due to the different screening supercurrents and pinned flux distributions^16,19,20,21^ (Supplementary Figs. S5–S7). The stray magnetic field of the superconductor was calculated using a 3D finite-element model based on the H-formulation of Maxwell’s equation^40,41^ (see Sect. 2 of Supplementary Information).

The micromagnetic calculations reveal a behavior that is in qualitative agreement with our experimental observations (Fig. 4a,b), namely an homogeneous switching field in the normal state of the YBCO (µ_0_*H*^z^_s_(*x*,*y*) ≈ 58 mT) and a gradient on *H*^*z*^_*s*_(x,y) within the superconducting state where the switching field increases from µ_0_*H*^z^_s_ ≈ 40 mT at the edges to µ_0_*H*^z^_s_ ≈ 48 mT at the center. The reversal of the magnetization occurs through nucleation and propagation of the magnetic domains. In the superconducting state, the nucleation mostly occurs at the edges and takes place at field values (µ_0_*H*^z^_s_ ≈ 40 mT) substantially lower as those obtained in the normal state (µ_0_*H*^z^_s_ ≈ 58 mT). This is due to the fact that the in-plane field components of the stray field of the SC are maximal (≈ 8 mT) at these positions easing the nucleation (Supplementary Figs. S6, S9), and due to the closure at the edges^42,43^ of the flux lines inside the superconductor (≈ 10 mT) which results in a local effective field larger than that applied one (Fig. 4c–f, Supplementary Fig. S7). On the other hand, the propagation depends on the effective out-of-plane field. With the chosen parameters for the disorder (see Sect. 3 of Supplementary Information for details), the simulations show that the domain propagation is locally activated when the magnetic field µ_0_*H*^z^(*x*,*y*) is above a threshold µ_0_*H*^z^_th_ ≈ 20 mT (horizontal dashed lines in Fig. 4c–f). In the normal state of the YBCO, the nucleation field (here, it is the switching field, 58 mT) is much larger than the field required for the propagation of the magnetic domains. Consequently, the magnetization reversal occurs over the entire film due to the immediate domain wall propagation following the random nucleation of domains. In contrast, in the superconducting state the propagation of the domains, nucleated at the edges, towards the center is hindered by the profile of *H*^z^_SC_ which leads to regions close to the center where *H*^z^(*x*,*y*) = *H*^z^_pulse_ + *H*^*z*^_sc_(*x*,*y*) falls below µ_0_*H*^z^_th_ ≈ 20 mT (Fig. 4d, top). After the field pulse, the pinned flux in the superconductor yields to an even lower effective field profile at remanence *H*^z^(*x*,*y*) = *H*^z^_sc_(*x*,*y*) that leaves the domain configuration mostly unaffected (Fig. 4d, bottom). This stability is overcome by further increasing *H*^*z*^_pulse_, which leads to exceeding the propagation field (*H*^*z*^(x,y) > µ_0_*H*^*z*^_th_ ≈ 20 mT) a bit further into the structure (Fig. 4e) and thus to a stepwise propagation of reversed domains (Fig. 4e). At µ_0_*H*^*z*^_pulse_ ≈ 48 mT, the effective field at the peak of the pulse is still below the propagation field µ_0_*H*^z^_th_ ≈ 20 mT nearby the square center (Fig. 4f, top). The magnetization switching within this region takes place at the end of the pulse due to the local increase of the effective field (Fig. 4f, bottom) related to the full flux penetration across the structure.

Experimentally, we observed that in the superconducting state a magnetic domain configuration with two concentric regions with roughly square-like shape and having relative antiparallel orientation of the magnetization direction can be imprinted, their relative size depending on *H*^*z*^_pulse_. The micromagnetic calculations have shown that, for a given *H*^*z*^_pulse_ range, the imprinted magnetic domain pattern is stable at remanence as long as *H*^*z*^_*sc*_(*x*,*y*) after the pulse is smaller than the propagation field µ_0_*H*^z^_th_. Imprint of similar magnetic domain patterns in other PMA systems is possible as long as this condition is also fulfilled. This can be achieved by proper selection and design of the PMA compound and/or by reducing the amplitude of *H*^*z*^_sc_ which is directly related to the critical current of the SC^19,20,21,44^. We note that the fine details about the switching process (nucleation field, propagation of inverse-magnetized domains and switching field profile) will depend on the full penetration field of the SC and to a minor extent on the sweep rate of the magnetic field pulse (see Sects. 4 and 5 in Supplementary Information).

The role played by $\overrightarrow{H}$_sc_ on the stabilization of imprinted spin textures in the absence of an external magnetic field is opposed in PMA and IMA systems. In PMA systems the condition for stability is that the effective field *H*^z^, after the imprinting pulse at T > T_C_, is kept below a threshold field. This condition can be fulfilled in both the superconducting (*H*^z^_sc_ < *H*_th_ − *H*^z^_ext_) and normal states (*H*^*z*^_*ext*_ < *H*_*th*_), thus allowing for the persistence of the imprinted domain distribution at T > T_C_. Contrarily, for IMA materials such as Py the stabilization does require the presence of in-plane field components *H*^x,y^_sc_(*x*,*y*) large enough to pin the generated magnetic pattern^10,27,28^. This precludes the persistence of the imprinted structures as temperature is raised, because the critical current of the superconductor and hence $\overrightarrow{H}$_sc_, decreases^27,21^ and eventually vanishes as T approaches T_C_, leading to a relaxation of the imprinted magnetic structure^27,28^.

**Figure 5:**
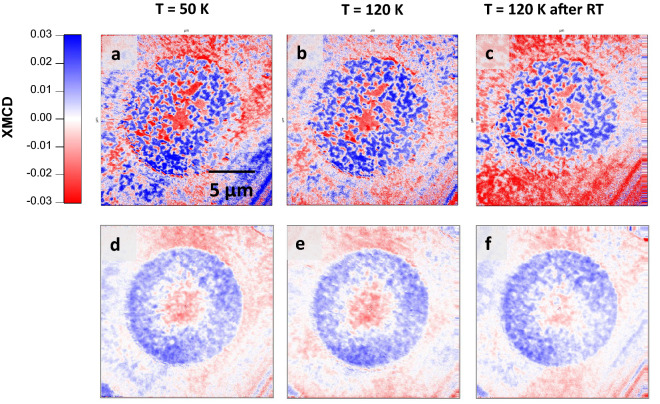
XMCD images for a given circular hybrid SC/FM structure of 13 µm diameter at H_ext_ = 0. (**a**) A magnetic domain configuration with two concentric regions with mostly antiparallel out-of-plane magnetic orientation (blue and red) has been imprinted at 50 K with the help of the SC stray field, see text for details about the magnetic history followed. The imprinted magnetic domain state is stable after increase T above Tc. (**b**) T = 120 K and (**c**) again T = 120 K after keeping the sample 20 min at room temperature (**d–f**) as in (**a–c**) where the XMCD images result from the averaging of the XMCD data obtained for 15 structures.

The possibility to imprint, with the help of the SC stray field, a complex magnetic domain distribution which remains after increasing the temperature above Tc in the studied PMA Co/Pt multilayers is experimentally demonstrated in Fig. 5 for hybrid SC/FM discs (13 µm dimeter). Figure 5a shows the initial state, that is a magnetic domain configuration imprinted at 50 K with the help of the SC stray field. The imprint, obtained after µ_0_*H*^*z*^_pulse_ =  + 100 mT followed by µ_0_*H*^z^_pulse_ =  − 65 mT shows a magnetic domain pattern formed by two concentric regions; a central one with downward magnetization direction and an outer one with upward orientation. As in the case of the square structure, the magnetic domain distribution of the outer switched region for the disc is not homogeneous (compare to Fig. 2). Its domain state is reminiscent of a worm-like domain pattern, which differs from disc to disc (see Sect. 6 in Supplementary Information). Increasing the temperature above the SC transition does not lead to significant changes neither in the magnetic domain distribution nor in the XMCD strength (Fig. 5b) even after keeping the sample at room temperature for 20 min (Fig. 5c). Similar results have been obtained for a total of 15 discs (Supplementary Figs. S13–S15). Panels d to f of Fig. 5 show the XMCD images resulting from averaging the XMCD images measured for the 15 structures at 50 K and 120 K (including the image after the excursion up to room temperature). The averaged images allow masking the fine domain structure details and highlighting the statistical distribution of switched/unswitched domains, which clearly show the two concentric regions described above. This result suggests that using optimized magnetic layers and patterning of the superconductor it might be possible to generate magnetic textures alike to chiral magnetic bubbles also termed bubble-skyrmions^45–49^.

A last comment regards the connection between the results described here and the previous work by Vlasko-Vlasov et al. (Refs.^50,51^) on the interplay between magnetic structure and vortices in superconducting Nb and ferromagnetic garnet hybrid films. These papers discuss the coupling between domains and vortices of the same polarity (magnetization parallel to vortex-magnetic field). This coupling has an effect of arresting the vortex lattice and as discussed in these works, also affect magnetization dynamics through deterministic changes in domain size. This concept, albeit close to our work, displays also clear differences. Importantly, the effect reported in our manuscript on the modification of the switching field of the Co/Pt ferromagnet triggered by the stray field of the superconducting flux of a High Tc superconductor YBCO, is remanent when temperature is increased above Tc (even reaching room temperature). This excludes that it results from the vortex-domain coupling scenario. Moreover, we did not observe appreciable differences in the size of magnetic domains above and below of the superconducting Tc. This also excludes that our effect is driven by the screening of the distribution of stray fields of the stripe domain structure of the ferromagnetic film theoretically discussed in the works of Bulaevskii^52,53^ and Chudnovsky^12^.

## Conclusion

In this article, we have shown the possibility to generate variable magnetization patterns in PMA Co/Pt multilayers by means of superconducting stray fields which are stable in the absence of an external magnetic field. The imprinted domain distributions can be reversed, modified (the relative size of inner- and outer-regions can be controlled) and erased by following a proper magnetic field history. The imprinted patterns, as well as the mechanisms behind their formation and stabilization are different as compared to those observed for compounds with in-plane magnetic anisotropy. In particular, the stabilization of the domains for PMA systems eased by their large coercivity and remanence, allows to keep the imprinted pattern almost unaltered for T > T_C_ as opposed to the results reported for IMA systems like Py. This allows the possibility of having non-trivial magnetic domain configurations at room temperature after their imprint below the SC transition temperature.

The use of SC structures to obtain complex magnetic domain configurations in PMA compounds is applicable to other ferromagnetic or ferrimagnetic materials, provided that the superconducting field at remanence does not allow the propagation of the nucleated domains. Optimized magnetic layers and proper selection of the SC properties (critical current) should allow tailoring the structure of domains to a larger extent. While the imprint is easily scalable to larger dimensions^25^ decreasing its size might not be feasible when reaching proportions comparable to characteristic magnetic penetration lengths of the superconducting compound. Possible applications of the reported effect include controlling magnetization patterns in FM insulators of micrometer size which holds promise in the established field of cavity optomechanics^54^ or in the new field of cavity optomagnonics^55^. Indeed, the possibility of employing micron-size magnetic textures in these areas has been recently proposed by Proskurin et al. (Ref.^56^) and Graf et al. (Ref.^57^). The tunability of the magnetization texture by means of magnetic field pulses would be an asset as the strength of the photon-phonon or photon-magnon coupling could be controlled^57^.

## Methods

### PEEM imaging

X-ray PEEM is a magnetic and element selective technique with a resolution of ca. 30 nm. Unlike many other techniques (e.g. magnetic force microscopy), X-ray PEEM delivers direct information about the magnetization, and the element selectivity guarantees that the recorded magnetic information comes only from the element under investigation. Magnetic sensitivity arises from the difference in absorption of circularly polarized radiation with left and right helicity from a magnetic element^58^.

Experiments were done at the PEEM station at the UE49/PGMa beam line of the synchrotron radiation source BESSY II of the Helmholtz-Zentrum Berlin^59^. The angle of incidence of the incoming radiation with respect to the sample surface was of 16°, which ensured a sizable projection (28%) of the out-of-plane magnetization of the Co/Pt multilayer along the beam propagation direction (see Fig. 1a), which gives rise to the XMCD signal.

Magnetic imaging was always performed in zero external field after a magnetic field pulse. The maximum pulse amplitude was µ_0_*H*^*z*^_*pulse*_ =  ± 100 mT with a pulse duration of 0.5–1 s and increasing/decreasing field rates of 10 mT s^−1^. Images with a 25 µm field of view were collected at the Co *L*_3_-edge (777.55 eV) for incoming circularly polarized radiation with right (σ^+^) and left (σ^−^) helicity (Fig. 1a), respectively. A total of 30 images, each with a 3 s. integration time, were collected per helicity. Each image was normalized to a bright field image and drift corrected before their averaging. The XMCD images were obtained as (σ^-^ − σ^+^)/(σ^-^ + σ^+^) where σ^+^ and σ^−^ were the averaged images for right and left circular polarized radiation, respectively.

## Supplementary Information

Supplementary Information 1.

Supplementary Video 1.

Supplementary Video 2.
