# Supplementary material for: Superconducting imprint of magnetic textures in ferromagnets with perpendicular magnetic anisotropy
Source: Sci Rep. 2021 Oct 21;11:20788. doi: 10.1038/s41598-021-99963-w (PMC8531309; doi:10.1038/s41598-021-99963-w)
Supplement: Supplementary file 1 — Supplementary Information 1. [file 41598_2021_99963_MOESM1_ESM.pdf]

Supplementary Information

Superconducting imprint of magnetic textures in ferromagnets with perpendicular magnetic anisotropy.

A. Sander[1, †], G. Orfila[2, †], D. Sanchez-Manzano[2], N. Reyren[1], M. A. Mawass[3], F. Gallego[2], S. Collin[1], K. Bouzehouane[1], K. Höflich[3,‡], F. Kronast[3], F. Grilli[4], A. Rivera-Calzada[2], J. Santamaria[2], J.E. Villegas[1] and S. Valencia[3,*]

[1]*Unité Mixte de Physique, CNRS, Thales, Université Paris-Saclay, 91767, Palaiseau, France*

[2]GFMC. Dept. Fisica de Materiales. Facultad de Fisica. Universidad Complutense 28040 Madrid*, Spain*

[3] *Helmholtz-Zentrum Berlin für Materialien und Energie, Albert-Einstein-Str. 15, 12489 Berlin, Germany*

[4] *Karlsruher Institut für Technologie, Institut für Technische Physik, 76344 Eggenstein-Leopoldshafen, Germany*

†These authors contributed equally to this work.

‡Present address: *Ferdinand-Braun-Institut GMbH Leibnitz-Institut für Höchstfrequenztechnik, Gustav-Kirchhoff-Str. 4, 12489 Berlin, Germany*

*Corresponding author: sergio.valencia@helmholtz-berlin.de

1. Characterization of hybrid SC/FM structures

The as-grown YBCO film is decorated, as typically found in thin YBCO films, by the presence of CuO precipitates spaced several microns (Fig. 1c of main manuscript). The YBCO grows around screw dislocations which for the thickness range of these experiments give rise to micron size square pyramids with flat and continuous terraces, see Figure S1a. The deposition of a FM layer by means of sputtering covers well these terraces with flat and non-granular surfaces (Figure S1b) confirmed by the presence of large magnetic domains (Fig. 1d within manuscript). XMCD-PEEM images of the domain structure of Co/Pt grown on YBCO (Fig. 1d) are very similar to that measured by magnetic force microscopy for Co/Pt grown o Silicon wafer. This demonstrates that the choice substrate (YBCO or Si) have no effect on the magnetic properties, which are the standard, well documented ones for Co/Pt multilayers.

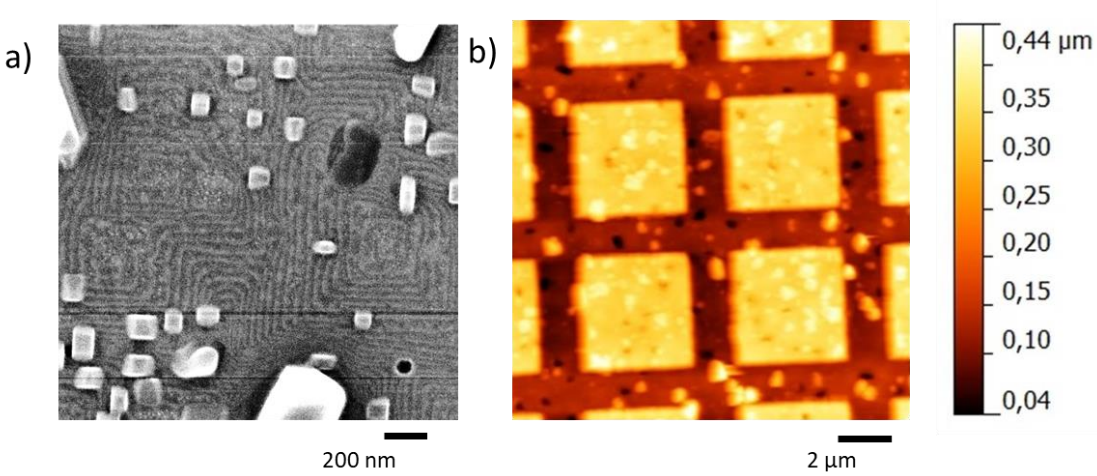

Figure S1 a) He ion microscopy images of a 250 nm thick YBCO film highlighting the presence of screw dislocations. b) AFM image of hybrid 5 µm X 5 µm SC/FM structures

The patterning process does not lead to a significant change of the superconductor (SC) transition temperature for the micrometric structures (not shown) in agreement with Simmendinger et al. (Ref. 1).

The effect of the CuO precipitates and the patterning on the coercivity, residual magnetization and magnetic domain structure, has been investigated by analyzing the full-field images of the XMCD-PEEM data depicted in Figure 2 for T > Tc. Full-field images are shown in Figure S2. The magnetic domain structure of (Co/Pt) is similar, within resolution, independently on whether the FM is deposited on top of the SC dot (central square) or directly on top of the substrate (panels a-e). The residual magnetization is almost 100% in both regions (panel f) as expected for systems with perpendicular magnetic

anisotropy. The "averaged" switching field for the FM on top of the SC is lower as compared to that on top of the substrate. This is likely to be due to the presence of CuO segregation on top of the YBCO square which acts as nucleation sites for reversed-magnetization domains.

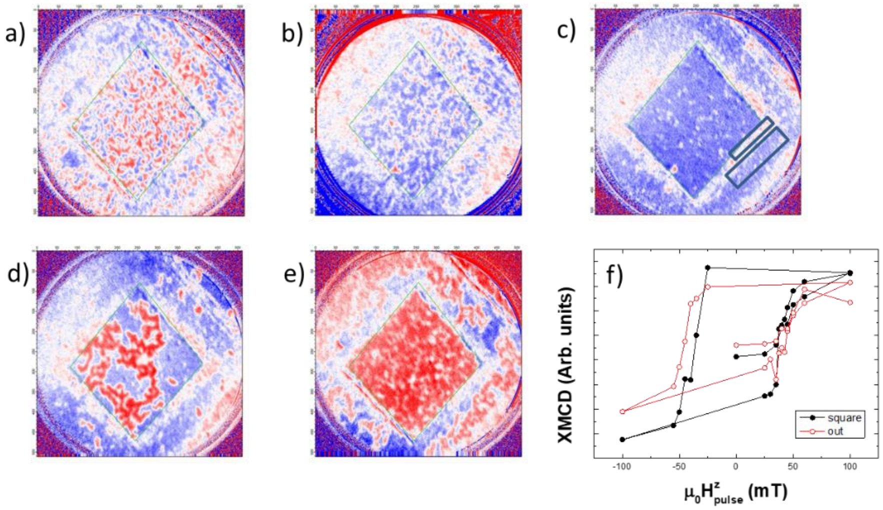

Figure S2 a)-e) XMCD-PEEM images obtained during a magnetic hysteresis cycle recorded at 120 K (T > Tc) after a ZFC. a) $\mu_0 H^z_{pulse}$ = +35 m**T**, b) $\mu_0 H^z_{pulse}$ = +50 mT, c) $\mu_0 H^z_{pulse}$ = -35 mT and d) $\mu_0 H^z_{pulse}$ = -50 mT Loops depicted in Figure R2f as function of Hpulse are obtained by integrating the XMCD signal for Co on top of the SC square (black) and directly on top of the substrate (red).

2. Magnetic field generated by a superconductor square

The magnetic field generated by the superconductor square ($\vec{H}_{sc}$) during and after the magnetic field application has been obtained by a 3D finite-element method based on the H-formulation of Maxwell's equations implemented in the finite-element software package Comsol Multiphysics[2,3]. The model solves Faraday's equation using a nonlinear resistivity for the superconductor material[4]

$$\rho(J) = \frac{E_c}{J_c}\left|\frac{J}{J_c}\right|^{n-1} \qquad \text{Eq. (1)}$$

where *Ec* is a threshold electric field, *Jc* is the critical current of the superconductor, and *n* a power index describing the steepness of the superconducting transition. Eq. (1) approximates Bean's critical state model[5] for sufficiently large values of n. For the simulations used for this work, the following values were used: Ec=10^{-4} V/m, J_c=10^{11} A/m^2 and n=25.

Simulations have been done for a squared superconductor structure with dimensions akin to those of the real system, i.e. 13 µm X 13 µm X 130 nm. The superconducting parallelepiped is surrounded by a cubic air domain. The external magnetic field is applied as a Dirichlet boundary condition on the face of the cube along the z axis. On the symmetry planes parallel and perpendicular to

the external field, conditions of magnetic insulation and perfect magnetic conduction are respectively applied. The air is modeled as a material with very large electrical resistivity (1 Ωm). The superconducting and air domains are meshed with hexahedral and tetrahedral elements, respectively. In particular, the superconducting domain is meshed with a grid of 24x24x3 elements (See Figure S3).

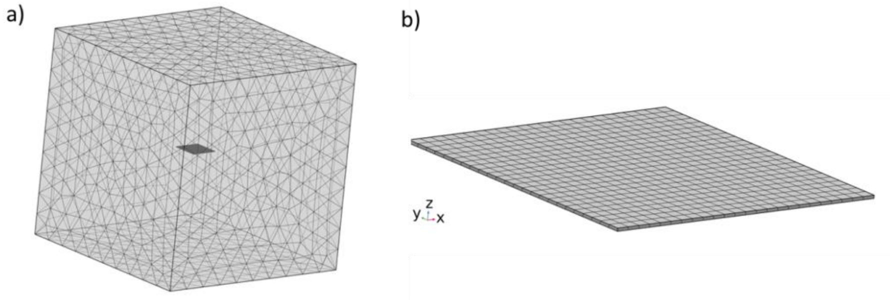

Figure S3 View of the simulated geometry and discretization. The external magnetic field is applied in the z direction. Top: Complete simulated geometry of a superconducting thin parallelepiped surrounded by a cubic air domain. The air domain is meshed with tetrahedral elements. Bottom: Detailed view of the superconducting domain, meshed with hexahedral elements.

The magnetic field history followed for the simulations was the following; after a pulse of $\mu_0 H^z_{pulse}$ = +100 mT we applied successive magnetic field pulses in -4 mT steps up to $\mu_0 H^z_{pulse}$ = -100 mT. A constant $\mu_0 dH/dt$ of 20 Ts^{-1} was used. For the sake of comparison with the experimental data we have inverted the sign convention within the main text and supplementary material i.e. H^z_{pulse} ➔ - H^z_{pulse} and $\vec{H}_{sc}$ ➔- $\vec{H}_{sc}$. Graphs within the main text and those which follow here do already consider this sign convention.

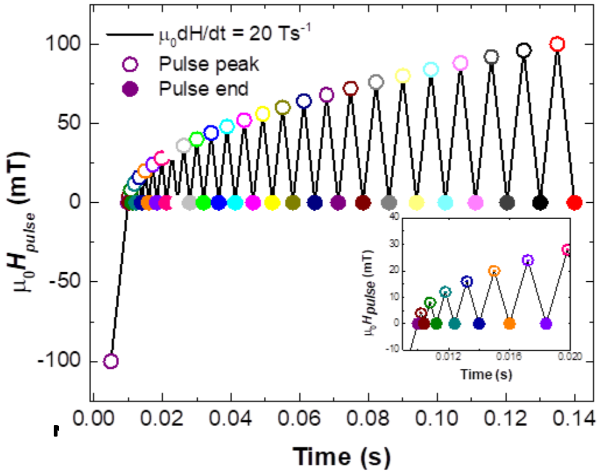

Figure S4 Magnetic field history followed for the calculations of the superconducting magnetic field components. Open dots at pulse peak and filled dots at pulse end. Inset shows detail for time up to 0.020 sec.

Error! Reference source not found. and **Error! Reference source not found.** show 2D maps of the computed y component of the supercurrent (J) and x and z components of $\vec{H}_{sc}$, respectively. $J^x(x,y)$ and $H^y_{sc}(x,y)$ can be obtained by a rotation of 90° of $J^y(x,y)$ and $H^x_{sc}(x,y)$. Top and bottom rows correspond to maps obtained at the peak of the magnetic field pulse (open dots in Figure S4) and at pulse end (filled dots in Figure S4). At remanence, both the components of the supercurrent and the superconducting field change with H^z_{pulse} strength. While the supercurrent (Fig. S5) seems to saturate at about 40 mT, the Z-component of $H^z_{sc}(x,y)$ still changes with H^z_{pulse}. The latter is clearly seen at the center of the square where we see that $H^z_{sc}(x,y)$ is different at 'pulse end' (filled dots) for $\mu_0 H^z_{pulse}$ = 40 mT, 44

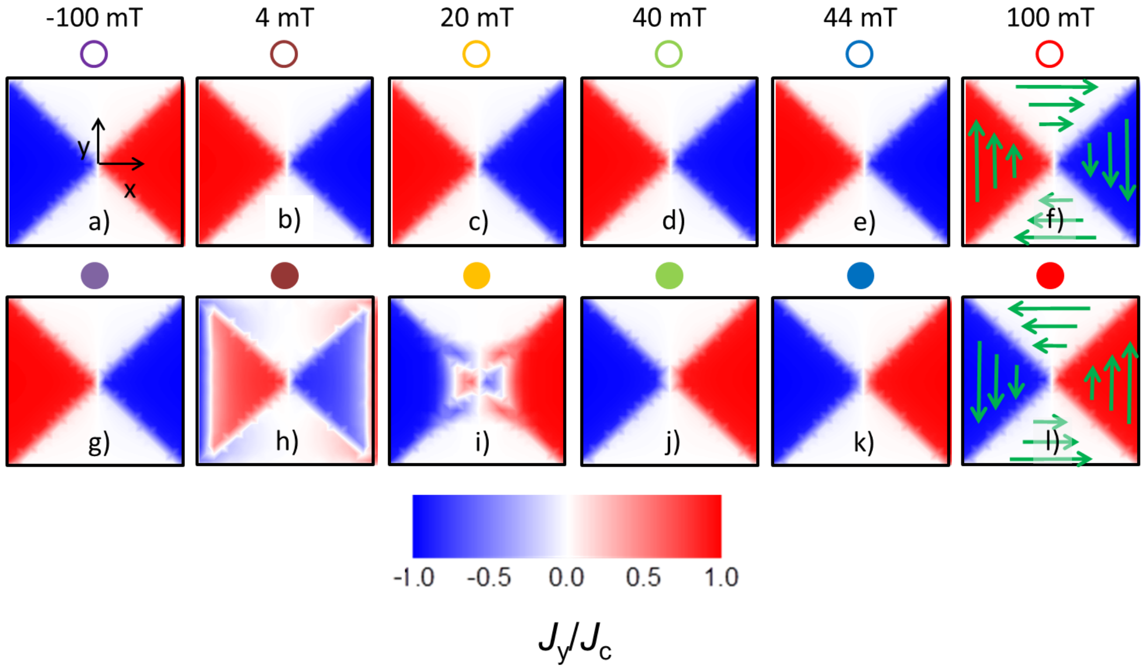

Figure S5 Two dimensional maps of the y component of the supercurrent normalized to the critical current (Jc) at selected H^z_{pulse} from magnetic history of figure S4, a-f) at peak pulse and g-l) at pulse end. Circles and ring on top of the panels indicate their position in Figure 2. The normalized x-component of the superconducting current can be obtained by a 90° rotation of Jy/Jc. Green arrows in panels f) and l) indicate the direction of current flow.

mT and 100 mT, panels j, k and l, respectively.

The magnetization of a SC structure vs an external out-of-plane magnetic field follows a characteristic hysteretic loop[6]. Full remanence of the magnetization within the SC is achieved for out-of-plane external fields $H^z_{ext} > 2H_p$ where H_p is the so called full penetration field[6,7]. Unless explicitly mentioned, from now on we will discuss the supercurrent and superconducting field components at

'pulse end' (colored filled dots in Figures S4-**Error! Reference source not found.**). The full penetration field describes the field for which at H^z_{ext} = 0 the supercurrents penetrating from the edges reach the center of the structure. As seen in figure S5g, after a -100 mT initial pulse the current flows clockwise. Increasing H^z_{pulse} leads to the penetration at the edges of supercurrents with counterclockwise sense of motion (Figs. S5h). For larger H^z_{pulse} currents extend towards the center (Figs. S5i). The counterclockwise current fully penetrates the structure for H^z_{pulse} between 20 and 40 mT (Fig. S8). On the other hand, $\vec{H}_{sc}$, and in particular H^z_{sc} saturates at larger fields i.e. for $\mu_0 H^z_{pulse}$ between 44 mT and 100 mT (Fig. S7k and S7l).

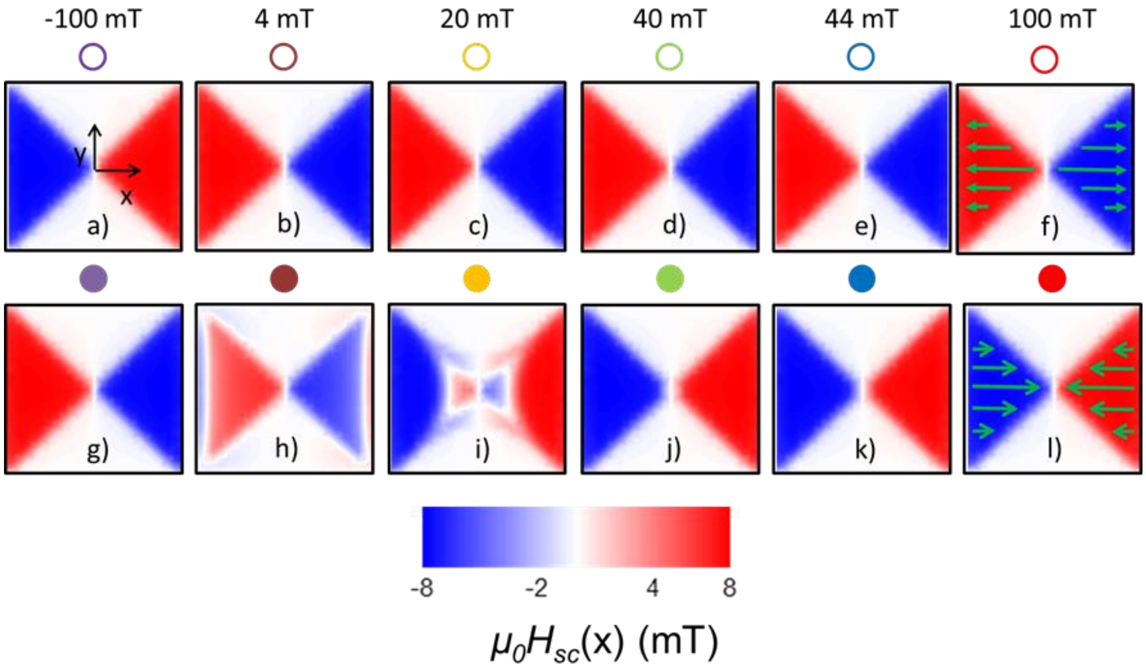

Figure S6 Two dimensional maps of the x component of the field generated by the superconductor at selected H^z_{pulse} from magnetic history of figure S4, a-f) at 'peak pulse' and g-l) at 'pulse end'. Circles and ring on top of the panels indicate their position in Figure 2. The map corresponding to the y component of the superconducting field can be obtained by a 90° rotation of H^x_{sc}(x). Green arrows in panels f) and l) indicate the direction of x field component

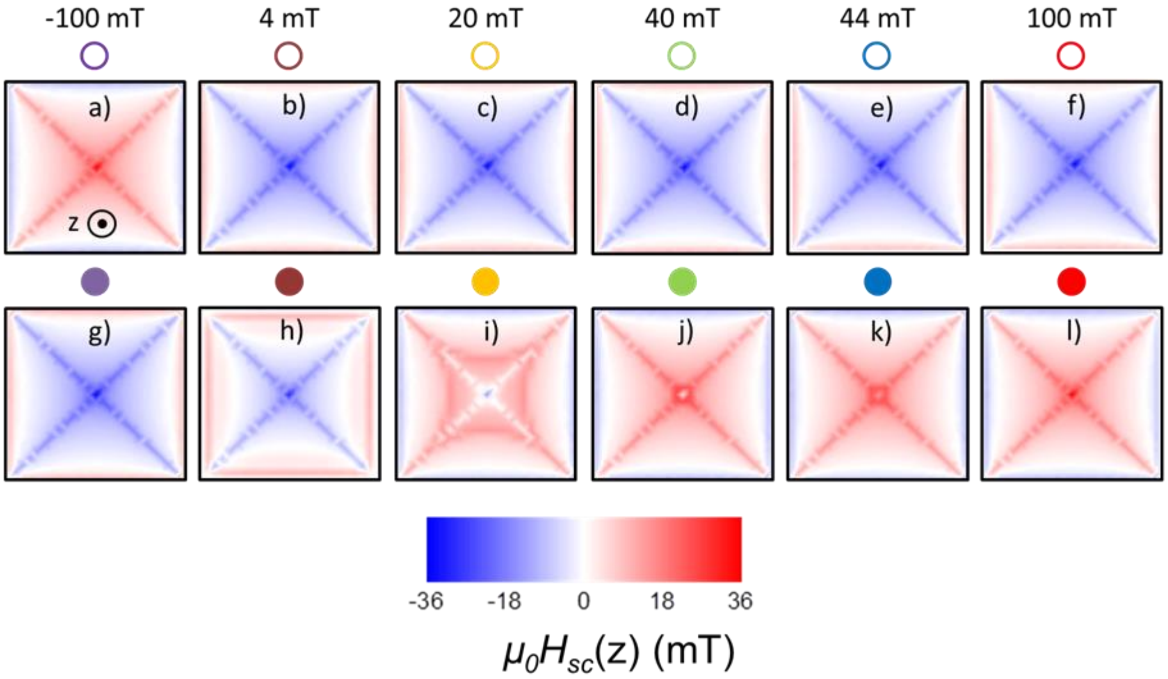

Figure S7 Two dimensional maps of the z component of the field generated by the superconductor at selected H^z_{pulse} from magnetic history of figure S4, a-f) at peak pulse and g-l) at pulse end. Circles and ring on top of the panels indicate their position in Figure 2.

Figure S8 shows the integration of the normalized y component of the supercurrent for x < 0 as function of H^z_{pulse} at ‘pulse end’ (squares). As comparison we also plot H^z_{sc} (0,0) at the center of the square (dots). While the current saturates at $\mu_0 H^z_{pulse}$ ≈ 24-28 mT, the z-component of the superconducting field at remanence (filled dots) does not saturate until ca. 52 mT. We deduce that $\mu_0 H_p$ is ca. 26 mT

We note that the field pulse range for which our micromagnetic simulations show a position dependent switching field for the Co/Pt multilayer is between 40 and 50 mT (Fig. 4b main text and orange bar in Figure S8). This field range stays within the field region for which $\vec{H}_{sc}$ of the superconductor is not saturated i.e. $H^z_s < 2H_p$. Hence, switching of the magnetization orientation for the Co/Pt multilayer takes place in a region where the superconducting field components do change with H^z_{pulse} (Fig. 4 within main text and Figs. S4 and S5). Full switching is achieved at field (ca. 50 mT) very close to that for which the superconducting field saturates.

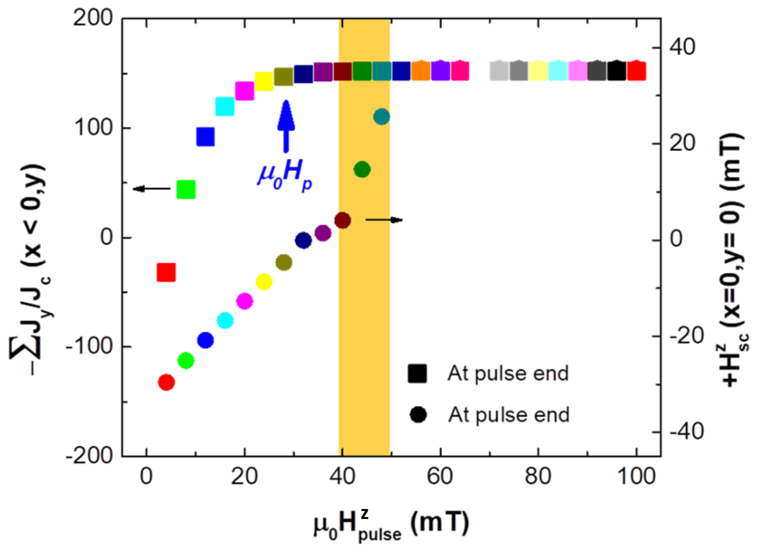

Figure S8 Left axis: Integration of the normalized y component of the supercurrent for half of the YBCO square (x <0) as function of H^z_{pulse} at pulse end. The current reaches full penetration (H_p) at $\mu_0 H^z_{pulse}$ ≈ 24-28 mT (blue arrow). Right axis: out-of-plane component of the superconducting fied at the center of the square as function of H^z_{pulse}. Magnetic saturation is reached at ca. 52 mT. Orange vertical bar shows the field range for which the micromagnetic simulations show switching of the magnetization for the hybrid $(Co/Pt)_5$/YBCO system, see Figure 4a and 4b within the main text.

For $H^z_{ext} > 2H_p$ the superconducting stray fields for the external field applied (‘pulse peak’) and at remanence (‘pulse end’) do not change and are the same except for a different sign. This can be seen for $\mu_0 H^z_{pulse}$ = +100 mT in Figure 4f within the main text (where we plot the effective field $H^z = H^z_{ext} + H^z_{sc}$) and in figures S6 and S7 (panels f and l).

3. Micromagnetic simulations

Micromagnetic simulations with MuMax3[8] have been performed considering an isolated 13x13 μm² square of 8-nm-thick magnetic ferromagnetic material with the effective parameters of the Co/Pt multilayer.

Among these parameters, we measured magnetization loops (parallel and perpendicular to sample plane) in twin samples using alternating gradient force magnetometry (AGFM) or SQUID and we evaluated the magnetization at saturation, M_s, and the effective out-of-plane anisotropy, K_{eff}. For the simulation, we hence considered the following measured Co properties: $M_s = 1.6$ MA/m (this value is large due to the proximity effect in Pt), the effective interfacial anisotropy $K_u = 1.8$ MJ/m^3, and the exchange $A = 12$ pJ/m. A is not directly measured in this study, but is estimated from previous studies[9]. To keep a manageable numerical problem (memory wise), we considered rather large cells (3 nm in plane, 8 nm out-of-plane), using a single effective magnetic layer. The parameters are adapted using an effective medium approximation as explained in, *e.g.*, Ref. [10], with the effective parameters (denoted by a '): $A' = A\,f$, $M_s' = M_s\,f$, $K_u' = K_u\,f - \mu_0 M_s^2/2\,(f - f^2)$, with $f = 0.6/1.6 = 0.375$ the ratio of the Co thickness over the total (Co|Pt) thickness. Because in ideal simulation the symmetry must be broken to allow nucleation (no thermal fluctuation is considered) and because small misalignment of the actual sample in the magnetic field is unavoidable, we considered a 0.5° angle between the applied field and the normal of the magnetic film (if not indicated otherwise). We know that our films, grown by sputtering at room temperature, are (1 1 1) textured with grains of typical lateral size of about 10 nm. This structure generates pinning that is modeled here as a 9% reduction of the exchange between grains, and 9% variation (standard variation) of the M_s, e.g. mimicking thickness variation[11]. This grain structure is necessary to stabilize the intermediate magnetization states that are observed. The behavior of the magnetic film is calculated by sequential minimization of the energy[12] using the external fields given by the finite element calculation of the fields generated by superconducting currents (see previous paragraph). The misalignment is simply modeled by an in-plane component of sin(0.5°) H^z_{ext}.

The micromagnetic calculations indicate that the reversal of the magnetization occurs through nucleation and propagation of the magnetic domains. Nucleation mostly occurs at edges and is favored by in-plane fields. In Figure S9, the displayed switching fields H^z as a function of in-plane field H^x demonstrates that H^z is reducing by much more than H_x. (For these micromagnetic simulations we used the same 8-nm-thick film, but we reduced its size to 1.3 x 1.3 μm².) On the other hand, the propagation depends on the external out-of-plane field. With the chosen disorder landscape (grain structure) and

misalignment of the field, the domain propagation starts above about 20 mT. One can note that the metastable states obtained during the magnetization reversal display a magnetization texture with small (few hundred nm wide) meandering domains, so-called labyrinthine or worm domains, with up/down domain ratio depending on the field history. In this system, a smooth micrometer long domain wall would be destabilized by the large dipolar energies, and the system reduces the total magnetic energy by forming these worm domains.

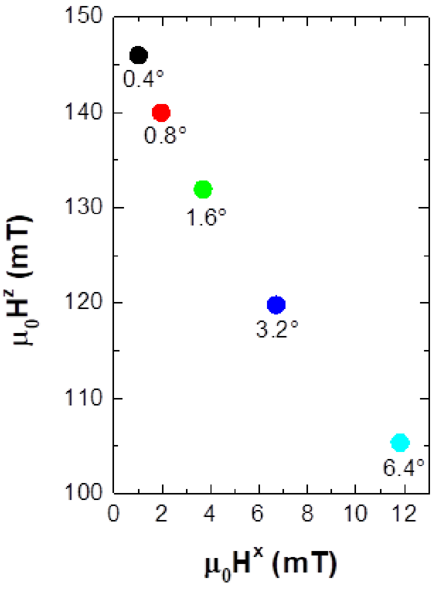

Figure S9 Switching field components for different misalignment angle (without superconducting field).

4. Effect of full saturation field on switching field profiles

The 3D finite-element calculations show that the field pulse range for which our micromagnetic simulations show a position dependent switching field for the Co/Pt multilayer stays within the field region for which $\vec{H}_{sc}$ of the superconductor is not saturated i.e. $H^z_s < 2H_p$, see Figure S8. In the following we address how the local switching fields depend on the relation between the magnetic field pulse range and the full penetration field (H_p) of the SC.

We considered three different scenarios:

i) "Real field" case (blue curve): Micromagnetic simulations are fed with the fields of the superconductor obtained after following the magnetic field history depicted in Figure S4.

ii) “High field” case (green curve): The magnetization of the SC is saturated for the full H_{pulse} range, i.e. $H_{pulse} > 2H_p$.

iii) “Low field” case (black curve): $H_{pulse} << 2\ H_p$ for all the H_{pulse} range of the experiments.

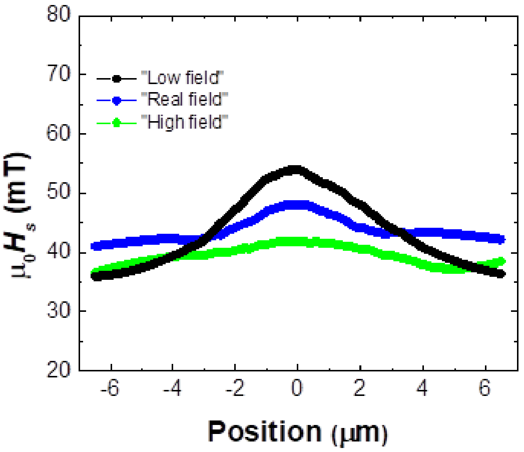

Figure S10 Line profiles of the switching field obtained by finite elements and micromagnetic simulations for the three cases described within the main text.

Figure S10 show the line profiles obtained for the switching field in the aforementioned cases, they all show a switching field which value depends on position. The line profile of the switching field obtained for the “high field” case (green) shows an effect smaller and with a smoother profile than that experimentally observed. The line profile obtained for the “low field” case shows the largest variation in switching field between the edges and the center of the structure and is the one which better resembles our experimental data. We note, however, that this case does not reflect the physics of our experiment; as an initial state we have assumed a saturated state for the magnetization of the SC, we have also assumed that $H_{pulse} << 2H_p$. The later means that the H_{pulse} range is not strong enough to modified the initial state. Experimentally this is contradictory; if the H_{pulse} range used is not able to modify the initial saturated state, the same H_p range would have not been enough to set this initial saturated state. The switching field profile for the “real” case is an intermediate case which reproduces fairly well our experimental observation that is, a switching field which varies faster close to the center of the structure than close to the edges.

The differences observed in the line profiles of the switching field for the 3 scenarios can be understand by considering their different H^z_{sc} profiles (Figure S11). As pointed out within the manuscript our initial simulations pointed out that “*... the domain propagation is locally activated when the magnetic field* $\mu_0 H^z(x,y)$ *is above a threshold* $\mu_0 H^z_{th} \approx 20$ *mT*”. Figure S11 shows the line profiles of H^z_{sc} at

pulse peak (dotted line) and at pulse end (continuous line) for the "high" and "low" field cases. Nucleation of reversed domains should take place at similar values for both "high" and "low" field cases as the z-profile of the stray field of the superconductor at the peak of the magnetic pulse is the same (dotted curves in Figure S11), see manuscript for details. After nucleation, domain propagation takes place. A reversal of the magnetization for the full structure is achieved once the z-component of the effective field is larger than the threshold field $\mu_0 H^z_{th}$ at every position (dashed line in Figure S11). For the "low field" case the field pulse necessary to fulfill this condition is ca. 57 mT. This value is the one necessary so that the center of the structure ($\mu_0 H^z_{sc}$ ≈ -37mT) reaches an effective field of 20 mT during the peak of the pulse (vertical blue line in left panel of Figure S11). For the high field case, a smaller field (ca. 40 mT) is enough to fully reverse the magnetization direction of the square structure. This is due to the fact that the central region of the square at pulse end always fulfills $\mu_0 H^z_{sc} > \mu_0 H^z_{t}$. A pulse of 40 mT is enough to bring above the threshold the effective field for the rest of the square during the pulse peak (vertical blue line in right panel of Figure S11).

These finite element and micromagnetic simulations strength the conclusions reported within the main manuscript and in addition highlight the importance of the relative size between H_{pulse} and H_p.

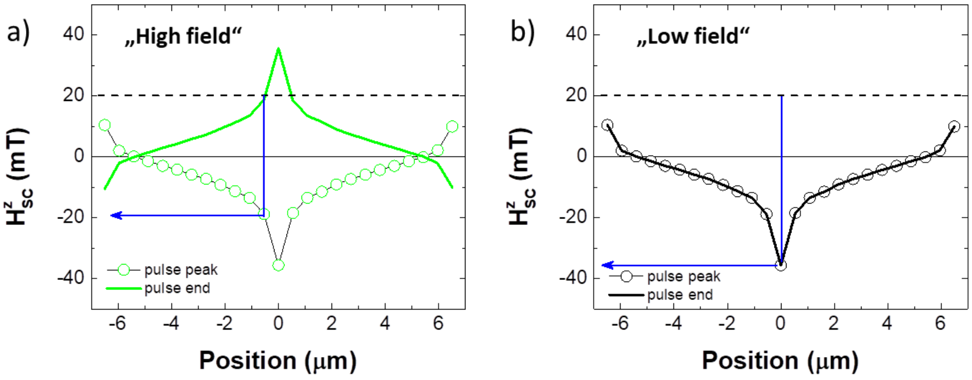

Figure S11 Line profiles of the z-component of the stray field of the superconductor at pulse peak (open dots) and pulse end (continuous line) for the "high" ($H_{pulse} > 2H_P$) and "low" ($H_{pulse} << H_P$) field scenarios, see text. The vertical blue line indicates the external field necessary so that the threshold field for domain propagation is fulfilled for the complete FM square.

5. Effect of pulse sweep rate and flux relaxation on imprinted magnetization distributions

The effect of the magnetic field pulse sweep rate on the imprinted magnetization distribution has been investigated by finite element calculations of the stray field of the superconductor. As argued within the main text, the z-component of the stray field of the superconductor ($H^z_{sc}(x,y)$) at pulse peak is

a key parameter to determine the extension of the switching. Consequently we have investigated how the z-component of the effective field, $H^z(x,y) = H^z_{pulse} + H^z_{sc}(x,y)$, changes depending on the switching field rate. Calculations have been restricted to 2D for the sake of keeping them within a reasonable time. Calculations have been done for infinitely thin disc of 13 μm diameter and for $\mu_0 H_{pulse} \approx 65$ mT (conditions alike to those reported in Figure 5 within main manuscript).

Figure S12 show radial plots of H^z profiles for different sweep rates at peak (left panel) and end (right panel) of the pulse. We observe that as the sweep rate for the magnetic pulse decreases:

i) At pulse peak (left panel):
   a. The effective field at the edges of the SC decreases slightly.
   b. The effective field at the center of the structure increases.

ii) At pulse end (right panel):
   a. The effective field at the center of the structure decreases slightly.

These observations leads to the following conclusion: decreasing sweep rates will require slightly larger fields for the nucleation of inversed magnetization domains and slightly smaller field pulses to reach full switching. The tendency shown by this “fast” simulations make us confident that these results can be reasonably extended to slower sweep rates (experiment sweep rate was $0.001 Ts^{-1}$). While it is true that the sweep rate have an effect on the nucleation and propagation of inversed-magnetizatio domains our results indicate that this effect is small, i.e. minor modification of the fields required for nucleation and full propagation.

In addition, magnetic relaxation of the SC remnant state will lead to a weakening of the effective field and thus will not modify the imprinted magnetic state.

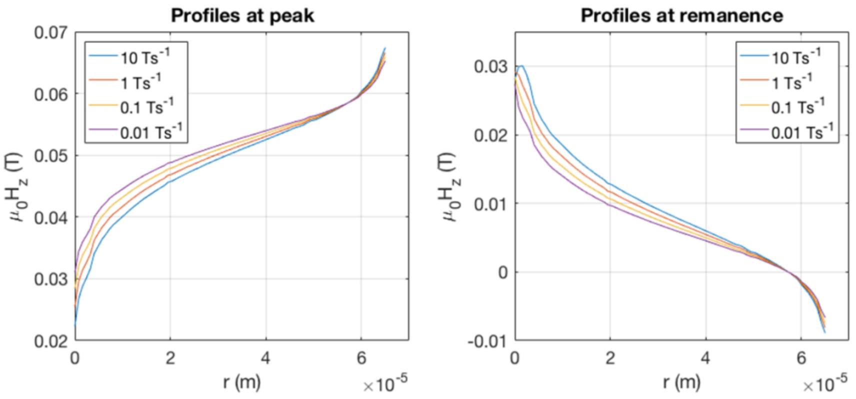

Figure S12 Radial plot of H^z at peak pulse (left) and pulse end (right) as function of pulse sweep rate.

The propagation of the inversed-magnetization domain towards the center takes mostly place at peak of the pulse as the z-component of the effective field reaches its maximum. Magnetic relaxation of the SC remnant state[13] will lead to a weakening of the effective field and thus will not modify the magnetic imprint.

6. PEEM on hybrid SC/FM circle structures

Figures S13, S14 and S15 show the XMCD images obtained for 15 hybrid structures with circular shape (diameter 13 μm).

A series of XMCD images as function of H^z_{pulse} were obtained for structure 1a (Figure S13 and Fig 5 within main text) in order to determine a proper magnetic field history leading to a magnetic domain structure with two concentric domains having antiparallel orientation (not shown). Top row of images of Figs. Figure ***S13***-Figure ***S15*** show the XMCD images obtained at 50 K for the 15 structures after the sample followed the determined magnetic field history (see main text). Images at 120 K (T > T_{sc}) where obtained after 30 minutes at the target temperature (middle of images on Figs. S13-S15). The magnetic domain pattern imprinted at 50 K is still clearly visible without appreciable changes at 120 K, small changes in XMCD contrast are attributed to differences in focusing conditions and beam position and

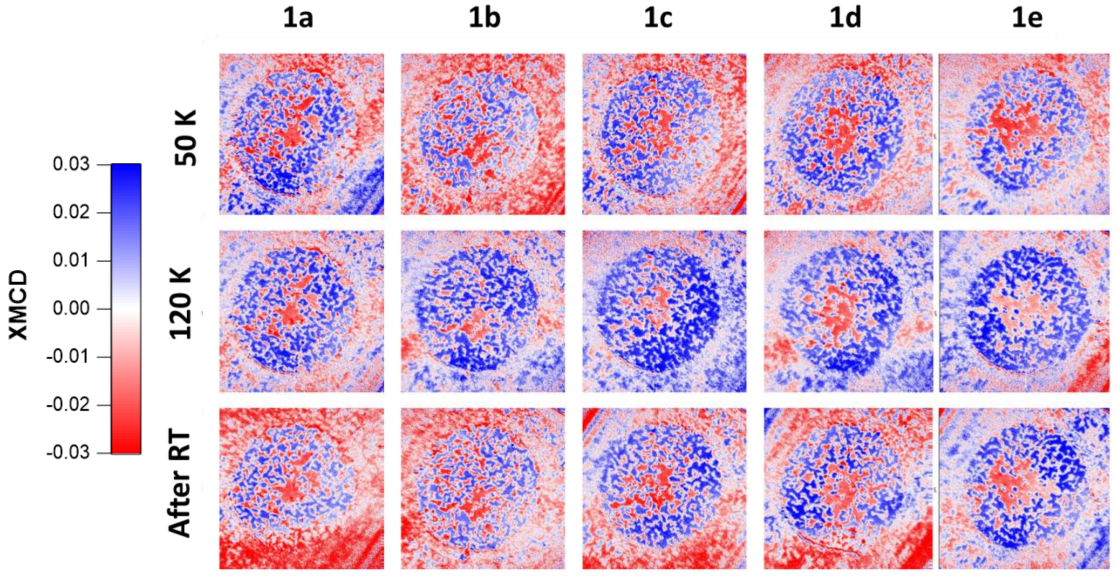

Figure S13 XMCD images obtained for hybrid SC/FM structures 1a to 1e after superconducting magnetic field imprint at 50 K (top row), after increasing T to 120 K (middle row) and after 20 min. at 300 K and cooling back to 120 k (bottom row).

beam drift during data acquisition. Increasing the temperature to room temperature leads to degassing of the sample and an increase of the pressure at the PEEM which preclude imaging at this temperature. We therefore obtained images at 120 K after keeping the sample at RT for 20 minutes. The obtained images are shown in the bottom row of images in Figs. S13 to S15. The magnetic domain texture does still resemble the one imprinted at 50 K.

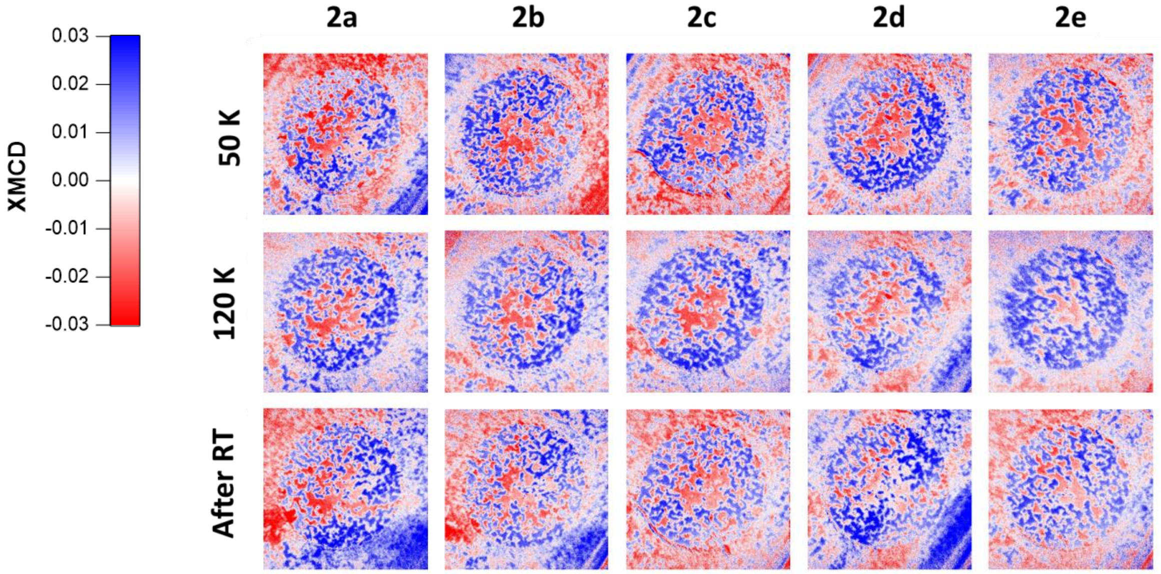

Figure S14 XMCD images obtained for hybrid SC/FM structures 2a to 2e after superconducting magnetic field imprint at 50 K (top row), after increasing T to 120 K (middle row) and after 20 min. at 300 K and cooling back to 120 k (bottom row).

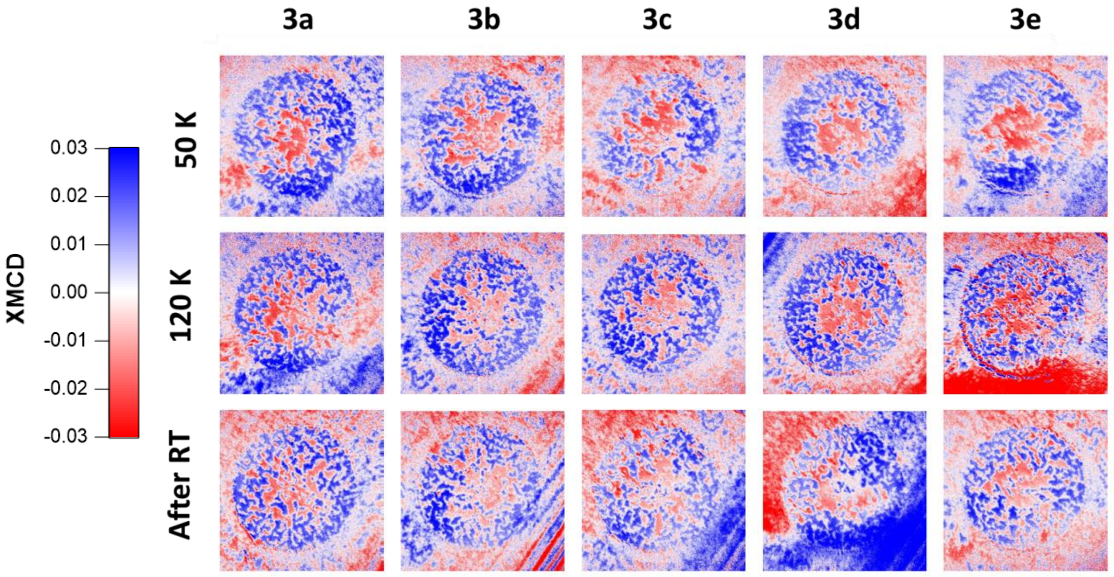

Figure S15 XMCD images obtained for hybrid SC/FM structures 3a to 3e after superconducting magnetic field imprint at 50 K (top row), after increasing T to 120 K (middle row) and after 20 min. at 300 K and cooling back to 120 k (bottom row).

References

1 Simmendinger, J. *et al.* Transmission x-ray microscopy at low temperatures: Irregular supercurrent flow at small length scales. *Physical Review B* **97**, 134515, doi:10.1103/PhysRevB.97.134515 (2018).

2 Brambilla, R., Grilli, F. & Martini, L. Development of an edge-element model for AC loss computation of high-temperature superconductors. *Superconductor Science and Technology* **20**, 16-24, doi:10.1088/0953-2048/20/1/004 (2006).

3 Grilli, F., Brambilla, R., Sirois, F., Stenvall, A. & Memiaghe, S. Development of a three-dimensional finite-element model for high-temperature superconductors based on the H-formulation. *Cryogenics* **53**, 142, doi:10.1016/j.cryogenics.2012.03.007 (2013).

4 Rhyner, J. Magnetic properties and AC-losses of superconductors with power law current—voltage characteristics. *Physica C: Superconductivity* **212**, 292-300, doi:https://doi.org/10.1016/0921-4534(93)90592-E (1993).

5 Bean, C. P. Magnetization of Hard Superconductors. *Physical Review Letters* **8**, 250-253, doi:10.1103/PhysRevLett.8.250 (1962).

6 Navau, C., Del-Valle, N. & Sanchez, A. Macroscopic Modeling of Magnetization and Levitation of Hard Type-II Superconductors: The Critical-State Model. *IEEE Transactions on Applied Superconductivity* **23**, 8201023-8201023, doi:10.1109/TASC.2012.2232916 (2013).

7 Brandt, E. H. Superconductor disks and cylinders in an axial magnetic field. I. Flux penetration and magnetization curves. *Physical Review B* **58**, 6506-6522, doi:10.1103/PhysRevB.58.6506 (1998).

8 Vansteenkiste, A. *et al.* The design and verification of MuMax3. *AIP Advances* **4**, 107133, doi:10.1063/1.4899186 (2014).

9 Fallon, K. *et al.* Quantitative imaging of hybrid chiral spin textures in magnetic multilayer systems by Lorentz microscopy. *Physical Review B* **100**, 214431, doi:10.1103/PhysRevB.100.214431 (2019).

10 Lemesh, I., Büttner, F. & Beach, G. S. D. Accurate model of the stripe domain phase of perpendicularly magnetized multilayers. *Physical Review B* **95**, 174423, doi:10.1103/PhysRevB.95.174423 (2017).

11 Leliaert, J. *et al.* A numerical approach to incorporate intrinsic material defects in micromagnetic simulations. *Journal of Applied Physics* **115**, 17D102, doi:10.1063/1.4854956 (2014).

12 Exl, L. *et al.* LaBonte's method revisited: An effective steepest descent method for micromagnetic energy minimization. *Journal of Applied Physics* **115**, 17D118, doi:10.1063/1.4862839 (2014).

13 Miu, L. *et al.* Origin of the plateau in the temperature dependence of the normalized magnetization relaxation rate in disordered high-temperature superconductors. *Physical Review B* **78**, 212508, doi:10.1103/PhysRevB.78.212508 (2008).
